# N1-Src Kinase Is Required for Primary Neurogenesis in *Xenopus tropicalis*

**DOI:** 10.1523/JNEUROSCI.3881-16.2017

**Published:** 2017-08-30

**Authors:** Philip A. Lewis, Isobel C. Bradley, Alastair R. Pizzey, Harry V. Isaacs, Gareth J.O. Evans

**Affiliations:** Department of Biology, University of York, York YO10 5DD, United Kingdom

**Keywords:** embryo, morpholino, neurogenesis, splicing, tyrosine kinase, *Xenopus*

## Abstract

The presence of the neuronal-specific N1-Src splice variant of the C-Src tyrosine kinase is conserved through vertebrate evolution, suggesting an important role in complex nervous systems. Alternative splicing involving an *N1-Src*-specific microexon leads to a 5 or 6 aa insertion into the SH3 domain of Src. A prevailing model suggests that N1-Src regulates neuronal differentiation via cytoskeletal dynamics in the growth cone. Here we investigated the role of n1-src in the early development of the amphibian *Xenopus tropicalis*, and found that *n1-src* expression is regulated in embryogenesis, with highest levels detected during the phases of primary and secondary neurogenesis. *In situ* hybridization analysis, using locked nucleic acid oligo probes complementary to the *n1-src* microexon, indicates that *n1-src* expression is highly enriched in the open neural plate during neurula stages and in the neural tissue of adult frogs. Given the *n1-src* expression pattern, we investigated a possible role for n1-src in neurogenesis. Using splice site-specific antisense morpholino oligos, we inhibited *n1-src* splicing, while preserving *c-src* expression. Differentiation of neurons in the primary nervous system is reduced in *n1-src*-knockdown embryos, accompanied by a severely impaired touch response in later development. These data reveal an essential role for n1-src in amphibian neural development and suggest that alternative splicing of C-Src in the developing vertebrate nervous system evolved to regulate neurogenesis.

**SIGNIFICANCE STATEMENT** The Src family of nonreceptor tyrosine kinases acts in signaling pathways that regulate cell migration, cell adhesion, and proliferation. Srcs are also enriched in the brain, where they play key roles in neuronal development and neurotransmission. Vertebrates have evolved a neuron-specific splice variant of C-Src, N1-Src, which differs from C-Src by just 5 or 6 aa. N1-Src is poorly understood and its high similarity to C-Src has made it difficult to delineate its function. Using antisense knockdown of the *n1-src* microexon, we have studied neuronal development in the *Xenopus* embryo in the absence of *n1-src*, while preserving *c-src*. Loss of n1-src causes a striking absence of primary neurogenesis, implicating n1-src in the specification of neurons early in neural development.

## Introduction

The Src family of 11 nonreceptor tyrosine kinases evolved to regulate key signaling pathways involved in cell adhesion, migration, and cell fate in multicellular organisms (Thomas and Brugge, 1997). Several Src family members, including C-Src, Fyn, and Yes, are enriched in the vertebrate nervous system with roles in the developing and mature brain and have been implicated in the pathology of neurological disorders (Grant et al., 1992; Maness, 1992; Zhao et al., 2000; Ohnishi et al., 2001; Kalia et al., 2004; Nygaard et al., 2014). Further complexity and specificity of C-Src signaling in the brain is conferred by neuronal-specific splicing to yield N1-Src or N2-Src (Brugge et al., 1985; Pyper and Bolen, 1990). The N-Src splice variants contain an additional 6 or 17 aa respectively in the SH3 domain, and are encoded by microexons situated between exons three and four of C-Src (Martinez et al., 1987). We and others have shown that N-Srcs have a higher constitutive kinase activity and an altered SH3 domain substrate specificity compared with C-Src (Dergai et al., 2010; Keenan et al., 2015). However, their *in vivo* substrates are unknown.

**Figure 1.**
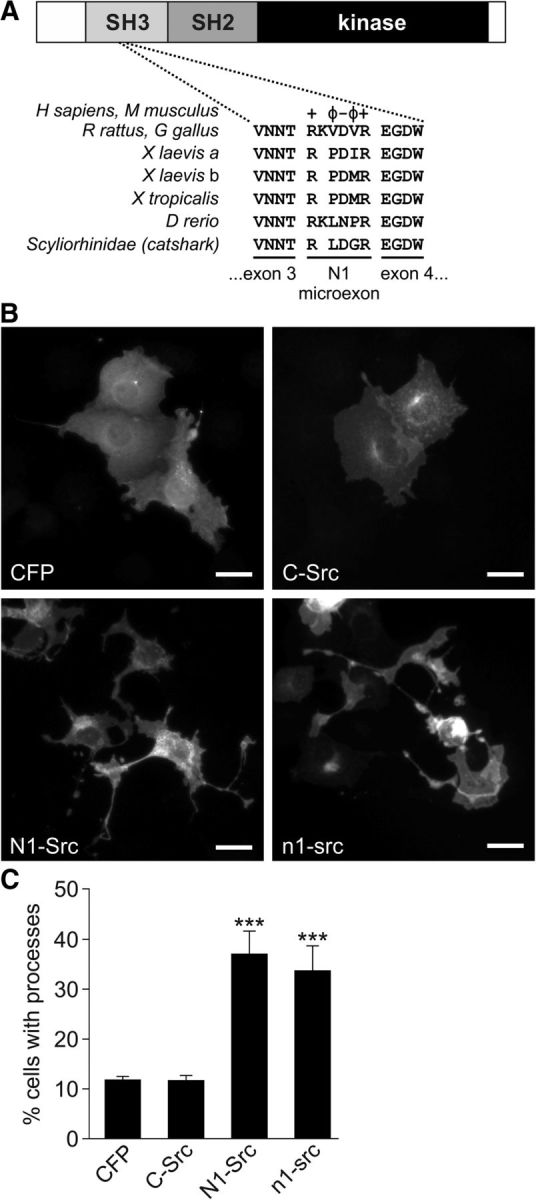
*Xenopus* n1-src elicits neurite-like processes in fibroblasts. ***A***, Amino acid alignment of the N1-microexon in mammalian, *Xenopus*, and fish species. +, Basic; −, acidic; Φ, hydrophobic amino acid sidechains. ***B***, Representative COS7 cells cotransfected for 4 d with Src-FLAG and CFP constructs. Cells were stained for Src (anti-FLAG) and CFP. ***C***, Quantification of process outgrowth in COS7 cells. Each process was defined as an extension longer than one cell soma diameter and <2 μm in diameter. Data are plotted as mean ± SEM (*n* = 3 independent experiments, analyzed by Kruskal–Wallis 2-tailed ANOVA; ***p < 0.001; Scale bar, 10 μm).

C-Src expression has been identified in a wide range of animal groups, including basal metazoans, such as sea sponges (Ottilie et al., 1992), but its neuronal splicing to yield N1-Src only appears in the vertebrate lineage (Fig. 1*A*; Levy et al., 1987; Martinez et al., 1987; Raulf et al., 1989) and N2-Src is restricted to mammals (Pyper and Bolen, 1990). Within the N1-Src microexon, there are minor differences in the length and sequence between vertebrate species. For example, a 6 aa N1-Src insert has been detected in brain tissue from the teleost fish *Xiphophorus* (Raulf et al., 1989), whereas the *c-src* locus of the diploid amphibian *Xenopus tropicalis* and the two pseudoallelic loci of allotetraploid *Xenopus laevis* contain 5 aa inserts (Collett and Steele, 1992). Identical 6 aa neuronal Src inserts are observed in N1-Src of chicks, rodents, and humans (Levy et al., 1987; Martinez et al., 1987). The appearance and conservation of a neural-restricted src isoform in the vertebrate lineage raises the intriguing possibility that n1-src function is related to the evolution and development of the complex vertebrate nervous system.

Previous studies in which N1-Src was overexpressed suggest N1-Src regulates neuronal morphology through cytoskeletal modifications affecting neurite outgrowth and axonogenesis (Worley et al., 1997; Kotani et al., 2007). However, no studies have thus far observed the development of the nervous system in the absence of N1-Src splicing. Here, we investigated n1-src function in the amphibian *Xenopus tropicalis*. We found that *n1-src* expression is localized to the dorsal ectoderm of the neural plate, which gives rise to the CNS during development. Using antisense morpholino oligos (AMOs), we have for the first time achieved specific inhibition of *n1-src* splicing in a vertebrate nervous system, without affecting *c-src* expression. The knockdown of *n1-src* caused abnormal touch responses in larval stage embryos, with a concomitant reduction in neuronal-specific tubulin (*tubb2a*)-positive neurons during primary neurogenesis. We propose that neuronal splicing of C-Src has evolved to be essential for vertebrate neurogenesis.

## Materials and Methods

### 

#### 

##### Subcloning of *Xenopus* n1-src.

A plasmid encoding C-terminal FLAG-tagged *Xenopus* n1-src (pFLAG-Xn1-Src) was generated by amplifying the *Xenopus laevis n1-src b* variant open reading frame from an IMAGE clone (ID: 5572523) with the following PCR primers incorporating 5′ BglII and 3′ KpnI restriction sites [this codes for an n1 insertion identical to that of *Xenopus tropicalis* n1-src, as determined by examination of the *Xenopus tropicalis* genome and sequencing of relevant reverse-transcription (RT) PCR products]: forward, 5′-AGATCTCTCTAGAACCATGGGTGCCACTAAAAGCAAGCCA-3′; reverse, 5′-GGTACCGTAGATCCAAGGTGTTCCCCAGGCTGGTACTG-3′.

Digested product was ligated into pEGFP-N1 (Clontech) in which the GFP tag was replaced with a FLAG tag (pFLAG). The pCS2+-Xn1-src-FLAG plasmid was generated by excising FLAG-tagged Xn1-src from pFLAG-Xn1-src with XbaI and ligating into XbaI-digested pCS2+. The preparation of pFLAG-C-Src and pFLAG-N1-Src was previously described (Keenan et al., 2015).

##### Fibroblast cell morphology assay.

Ten thousand COS7 fibroblast cells were plated onto 13 mm coverslips. Twenty-four hours after plating, cells were transfected with 1 μg of plasmid DNA using Ecotransfect (Oz Biosciences) according to the manufacturer's instructions. Cells were fixed 48 h after transfection in 4% paraformaldehyde, 4% sucrose for 20 min, and then permeabilized in 0.1% Triton 1% BSA and stained with primary antibodies [mouse anti-FLAG (M2), 1:1000; rabbit anti-GFP, 1:500] in 1% BSA in PBS for 2 h at room temperature. After three washes in PBS, secondary antibodies (anti-mouse Alexa Fluor 564 and anti-rabbit Alexa Fluor 488; Invitrogen) were applied at 1:500 in 1% BSA in PBS for 1 h in the dark. Coverslips were mounted on slides using Mowial mountant (10% Mowial, 25% glycerol in 0.1 m Tris, pH 8.5) containing 1 μg/ml DAPI. Images were acquired using a 40× objective on a Nikon TE200 epifluorescence inverted microscope using a RoleraXR CCD (QImaging) camera controlled by SimplePCI Software (Hamamatsu). The percentage of COS7 cells bearing neurite-like processes was measured. Processes were defined as being longer than the cell-soma diameter and having a width of <2 μm.

##### Embryological methods.

All work involving animals was approved by the Biology Ethics Committee, University of York, and performed under the relevant UK Home Office legislation. *Xenopus tropicalis* embryos were produced as previously described (Khokha et al., 2005; Winterbottom et al., 2010). Embryos were microinjected at the two-cell or four-cell stage and cultured at 22°C in MRS/9 + 3% Ficoll, before transferring to MRS/20 for long-term culture. The sequences for the splice-blocking AMOs (GeneTools) are as follows: AMO a (splice acceptor), 5′-GTCAGGTCTCCTATGGCACAGCATG-3′; AMO d (splice donor), 5′-GCCGCCGGATGGTCACATACCTCAT-3′.

The touch response assay was conducted on Stage 28 or 41 embryos, in which the stationary animal was lightly touched on its side with the tip of a pair of forceps. Nonresponding embryos were scored as those that did not immediately swim out of the field of view in response to the touch stimulus. Videos of the locomotive phenotypes were acquired using a JVC TK-C1381 camera and processed with ArcSoft ShowBiz software.

##### RNA extraction and semiquantitative RT-PCR.

Demembranated embryos or tissues dissected from male adult *Xenopus tropicalis* were flash-frozen on dry ice. Total RNA was extracted from tissues using Tri-Reagent and precipitated with 7.5 m LiCl/50 mm EDTA (Warrander et al., 2016). First-strand cDNA was synthesized from 1–3 μg of total RNA using random hexamer primers and Invitrogen SuperScript II Reverse Transcriptase according to the manufacturer's instructions (Warrander et al., 2016). Promega PCR Master Mix was used to amplify cDNA from the embryos at the different developmental stages, with *rpl8* used as a loading control. Primers used to detect gene expression were as follows: *rpl8* forward, 5′-GGGCTGTCGACTTCGCTGAA-3′; *rpl8* reverse, 5′-ATACGACCACCACCAGCAAC-3′; *c-src* forward, 5′-ATCTCGCACCGAGACAGACT-3; *c-src* reverse, 5′-CAGTCGCCTTCCGTGTTATT-3′; *n1-src* forward, 5′-ACTGTGACCTGACGCCTTTT-3′; *n1-src* reverse, 5′-CCTCATGTCAGGTCTCGTGT-3′.

##### *In situ* hybridization and β-galactosidase staining.

*Tubb2a* (*n-tubulin*) probe synthesis and *in situ* hybridization were carried as previously described (Winterbottom et al., 2010). A 19-mer digoxigenin end-labeled locked nucleic acid (LNA) probe was designed against the 15 base *n1-sr*c microexon sequence, with the addition of two bases from the flanking *c-src* exons. The probe with the following sequence was synthesized by Exiqon (Darnell et al., 2010). Locked nucleotides are indicated in bold: *n1-src* microexon probe 5′-**T**CC**C**TC**A**TG**T**CA*GGT****C*TCG-**3′.

It was confirmed that no off-target sequence identities of >12 nt were present in the *Xenopus tropicalis* genome. The LNA *in situ* hybridization was undertaken as previously described (Sweetman, 2011; Warrander et al., 2016). Briefly, demembraned embryos were fixed in 0.1 m morpholinopropanesulfonic acid (MOPS), 2 mm EDTA, 1 mm MgSO_4_ 3.7% formaldehyde. The hybridization and washes were performed at 57°C. Embryos were hybridized for 36 h with 20 nm LNA probe preabsorbed against tailbud stage embryos. Color was developed with BM Purple (Roche) substrate until diffuse purple staining was visible, at which point embryos were washed for 12 h. The staining and washing cycle was then repeated until strong specific staining was present. For lineage tracing, β-galactosidase mRNA synthesis, embryo injection, and enzyme staining was undertaken as previously described (Pownall et al., 1996). Both *in situ* and fixed phenotypes were imaged using a Leica MZ FLIII microscope (Leica), a SPOT 14.2 Color Mosaic camera, and SPOT Advanced software (Diagnostic Instruments).

##### Experimental design and statistical analysis.

The fibroblast cell morphology assay was conducted in triplicate with ≥60 cells analyzed per condition for each independent replicate. The experimenter was blind to the plasmid transfected in each condition. Statistical analysis of the data was performed with SigmaPlot software using a Kruskal–Wallis ANOVA and Dunn *post hoc* pairwise comparisons. The *Xenopus* embryo touch response behavioral assay was conducted in triplicate with 20–65 animals analyzed per condition for each independent replicate. Statistical analysis of the percentage of nonresponding embryos was performed with a one-way ANOVA and Tukey's *post hoc* pairwise comparisons. For the *tubb2b in situ* hybridizations, 39–55 embryos derived from four fertilizations were unilaterally injected with control or *n1-src* AMOs. The proportion of embryos with abnormal *tubb2b* staining on the injected side compared with the contralateral internal control was statistically analyzed in R with a χ^2^ test.

## Results

### The *Xenopus* n1-src splice variant promotes neurite outgrowth

We first investigated whether the activity of N1-src isoforms has been conserved during vertebrate evolution. There are minor differences in the length and sequence of the *n1-src* microexon between mammals, amphibians, and fish. However, the distribution of charged and hydrophobic residues is conserved (Fig. 1*A*). Overexpression of mammalian N1-Src (but not C-Src) was previously shown to elicit morphological changes in *Xenopus* kidney epithelial cells (Worley et al., 1997) and we therefore performed a similar assay to compare the biological activity of *Xenopus* n1-src and mammalian N1-Src (Fig. 1*B*,*C*). COS7 fibroblasts were cotransfected with soluble CFP (to aid the visualization of cell morphology) and a C-terminal FLAG-tagged Src construct (C-Src, N1-Src, or n1-src) or a vector control. We and others have previously shown that C-terminal fusion tags do not affect Src activity in cells (Sandilands et al., 2004; Keenan et al., 2015). We assayed cell morphology by quantifying the percentage of cells bearing neurite-like processes. In agreement with previous findings, C-Src did not elicit process outgrowth compared with the vector control, while approximately one-third of N1-Src-transfected and n1-src-transfected cells bore processes (Fig. 1*B*,*C*), suggesting that, despite sequence differences their N1-Src insertions, activities of the amphibian and mammalian N1-Src enzymes have been highly conserved during evolution.

### *n1-src* is expressed during neurogenesis

**Figure 2.**
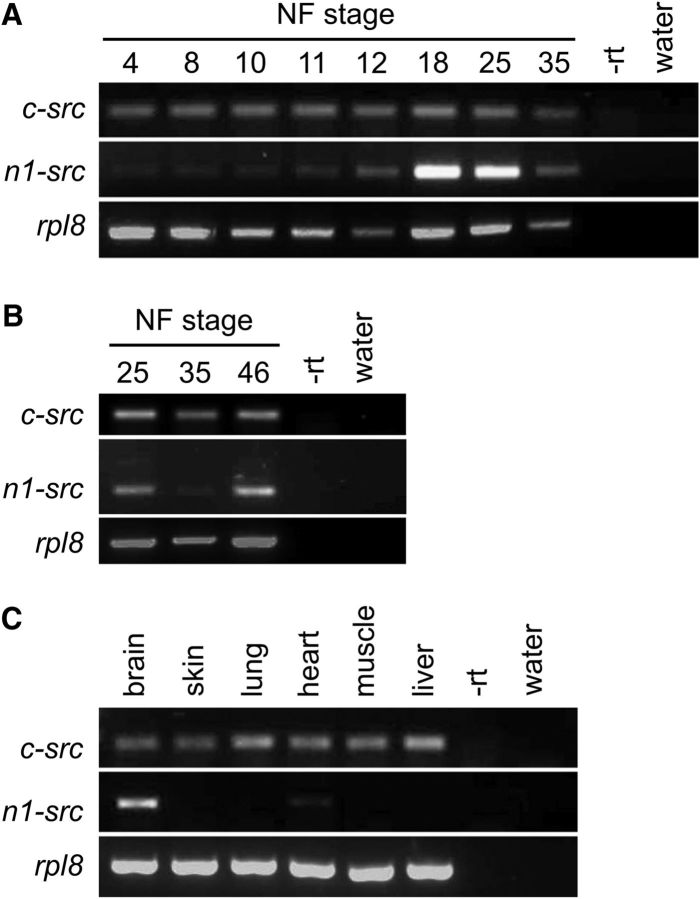
*n1-src* mRNA expression levels during *Xenopus tropicalis* development and in adult tissues. ***A***, RT-PCR analysis of *c-src* and *n1-src* mRNA expression levels from early cleavage Stage 4 to tailbud Stage 35. *rpl8* is used as a ubiquitously expressed loading control. −rt, No reverse transcriptase control; water, no template control. ***B***, RT-PCR analysis of *c-src* and *n1-src* expression levels during (Stage 25) and after (Stage 35) primary neurogenesis, and during secondary neurogenesis (Stage 46). ***C***, RT-PCR analysis of *c-src* and *n1-src* expression in a range of adult tissues.

We next examined the temporal expression of *Xenopus* n1-src during development. Using splice variant-specific PCR primer sets, we undertook RT-PCR analysis of *c-src* and *n1-src* expression from cleavage to early larval stages (Fig. 2*A*). Expression of *c-src* is relatively constant throughout early development. In contrast, *n1-src* expression is highly regulated over the same period. Before the onset of transcription from the zygotic genome at blastula Stage 8, only very low levels of maternally deposited *n1-src* mRNA are detected. Zygotic *n1-src* expression begins to rise at gastrula Stage 11, reaching its highest level at neurula Stage 18, and this is maintained through early tailbud Stage 25. However, by early larval Stage 35, expression has fallen dramatically. Figure 2*B* indicates that *n1-src* expression increases again at Stage 46, correlating with secondary neurogenesis of motor neurons, interneurons, and sensory neurons in the closed neural tube (Schlosser et al., 2002). Therefore, *n1-src* expression is maximal during phases of neurogenesis in the nervous system.

We also examined the expression of *c-src* and *n1-src* in adult tissues and found that the highest level of *n1-src* expression is within the adult brain. Outside the brain, we detected no *n1-src* expression, except at low levels in heart muscle (Fig. 2*C*).

### *n1-src* expression is enriched in the neural plate

**Figure 3.**
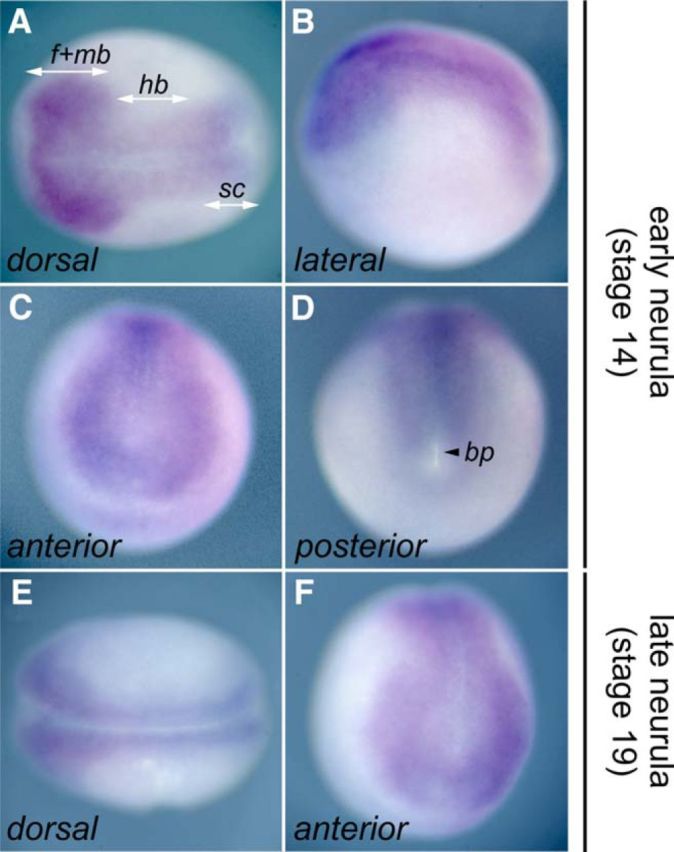
Expression pattern of *n1-src* during *Xenopus tropicalis* primary neurogenesis. *In situ* hybridization analysis of *n1-src* mRNA expression using a 19-mer digoxigenin end-labeled antisense probe directed against *n1-src*-specific sequence. ***A–D***, Early neurula Stage 14 embryos. ***E***, ***F***, Late neurula Stage 19 embryos. ***A***, Dorsal view, anterior to left. ***B***, Lateral view, anterior to the left. ***C***, Anterior view, dorsal to the top. ***D***, Posterior view, dorsal to the top. ***E***, Dorsal view, anterior to the left. ***F***, Anterior view, dorsal to the top; *f*+*mb*, presumptive forebrain and midbrain; *sc*, presumptive spinal cord; *bp*, blastopore.

To visualize the spatial expression pattern of n1-src in the developing embryo, we used a 19-mer LNA probe specific for the *n1-src* microexon sequence. Traditional antisense mRNA *in situ* probes are typically >∼150 bases in length and are unable to distinguish between the small sequence differences exhibited by the *c-src* and *n1-src* splice variants. Early-stage and late-stage neurula embryos were probed with a digoxigenin-labeled *n1-src*-specific LNA probe. In keeping with our RT analysis, we found that *n1-src* expression is highly enriched in the neural plate of neurula-stage embryos (Fig. 3). Our analysis indicates general expression of *n1-src* in cells of the neural plate at Stage 14 (Fig. 3*A*,*B*). Expression is fairly constant along the anteroposterior axis of the neural plate, with expression detected in cells of the presumptive forebrain, midbrain, and hindbrain regions, as well as the spinal cord (Fig. 3*A*,*C*,*D*). n1-src expression continues to be enriched in the neural plate as it narrows and rolls up to from the neural tube in late Stage 19 neurula embryos (Fig. 3*E*,*F*).

### Morpholino-mediated knockdown of *Xenopus* n1-src disrupts the touch response of embryos

**Figure 4.**
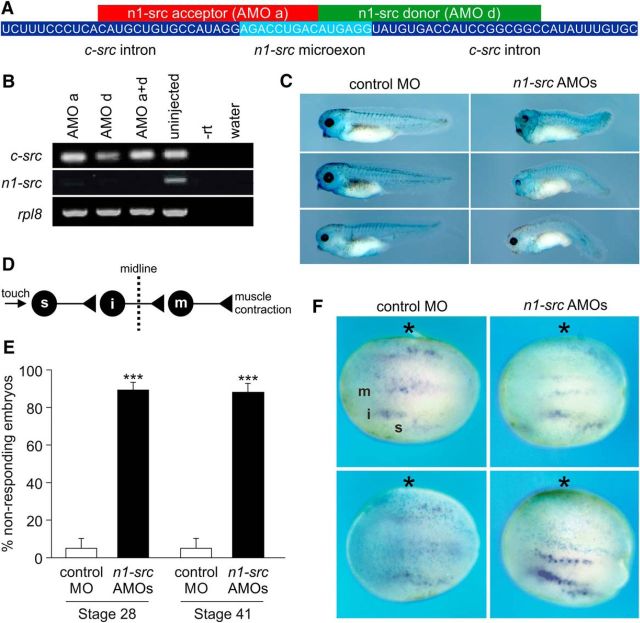
Abnormal touch response and primary neurogenesis in n1-src-knockdown embryos. ***A***, Diagram showing the sequences and corresponding RNA target sequences of the splice acceptor (AMO a) and donor (AMO d) splice-blocking AMOs. ***B***, RT-PCR analysis of *c-src* and *n1-src* mRNA expression at Stage 16 in control uninjected embryos and embryos injected with a total of 20 ng AMO a, AMO d, or AMO a+d. *rpl8* is used as a ubiquitously expressed loading control. −rt, No reverse transcriptase control; water, no template control. ***C***, Representative phenotypes of embryos at larval Stage 41 bilaterally injected at the two-cell or four-cell stage with 10 ng total of a standard control MO or the AMO a+d combination. Embryos were coinjected with 100 pg of nuclear β-galactosidase and subsequently stained with X-gal (light blue) to demonstrate successful injection targeting. ***D***, Diagram of embryo touch reflex. Touching the skin stimulates Rohon–Beard sensory neurons (s), which synapse onto commissural interneurons (i) that activate contralateral motor neurons (m), leading to muscle contraction. ***E***, Quantitation of touch-response phenotype of the same embryos at larval Stage 28 and 41 bilaterally injected at the two-cell or four-cell stage with 10 ng total of a standard control MO or the AMO a+d combination. Data are plotted as mean ± SEM (*n* = 3 independent experiments, analyzed by 1-way ANOVA with Tukey's *post hoc* test, ***p < 0.001, control MO vs n1-Src AMO). ***F***, *In situ* hybridization analysis of *tubb2b* expression in differentiating primary neurons of open neural plate Stage 14 embryos unilaterally injected with 5 ng total of a standard control MO or the AMO a+d combination. The injected side shows faint blue nuclear staining with the β-galactosidase lineage tracer, and is indicated with a black asterisk. Anterior is to the left. m, Motor neurons; i, interneurons; s, sensory neurons.

MOs, which are nucleic acid analogs with a modified backbone chemistry, are able to hybridize to target RNA in a highly specific, sequence-dependent fashion. AMOs are able to knock down gene function in a number of systems (Eisen and Smith, 2008). Typical knockdown strategies use AMOs to block translation or nuclear pre-mRNA processing. AMOs targeted to splice acceptor and donor sites in pre-mRNAs have been used to block normal splicing events, leading to the formation of aberrant mRNAs containing intron sequences, thus disrupting the protein coding information normally found in the mature mRNA. AMOs have also been successfully used to induce exon skipping (Goyenvalle et al., 2010; Kang et al., 2011). Here we use this approach to block splicing involving the *n1-src*-specific microexon. Nonoverlapping AMOs targeted to the splice acceptor (AMO a) and donor (AMO d) sites of the *n1-src* microexon were designed (Fig. 4*A*). *n1-src* AMOs were delivered to the cells of the embryo by microinjection. In contrast to uninjected control embryos, we detected no *n1-src* expression in embryos injected with a combination of AMO a and AMO d. Furthermore, injection of AMO a or d alone also effectively blocked *n1-src* expression (Fig. 4*B*). Consistent with an effect on exon skipping, the expression of *c-src* was unaffected by the AMOs. We conclude that AMOs represent highly specific tools for investigating the function of the *Xenopus* n1-src isoform in early development.

Injection of a standard control MO has little effect on the phenotype of larval Stage 41 embryos, whereas injection of the AMO a+d mixture leads to a mild, but highly penetrant phenotype, which is characterized by shortening and/or kinking of the tail, and variable disruption to the pigmented retina of the eye (Fig. 4*C*). To assess the function of the primary nervous system in *n1-src*-ablated embryos, we applied a touch stimulus to the side of larval-stage embryos, which normally elicits a dart response. The neuronal circuitry for the touch reflex (Fig. 4*D*; Movies 1, 3) is well characterized and comprises Rohon-Beard sensory neurons, which activate commissural interneurons that in turn synapse onto contralateral motor neurons to stimulate muscle contraction, propelling the embryo away from the stimulus (Boothby and Roberts, 1995; Li et al., 2003; Roberts et al., 2010; Fig. 4*D*; Movies 1, 3). We tested the same embryos at developmental Stages 28 and 41 (before and during the onset of myelination) and the dart response was commonly abnormal or absent in *n1-src* AMO a+d-injected embryos compared with controls (Fig. 4*E*; one-way ANOVA, *F* = 96.3, df = 3, *p* = 1.28 × 10^−6^). These embryos instead frequently displayed an uncoordinated twitch or spasm response, indicating abnormal development of the neural circuitry necessary for the dart response (Fig. 4*D*; Movies 2, 4).

Movie 1. Normal touch response in Stage 28 *Xenopus tropicalis* embryos. Real-time video showing the normal touch response of Stage 28 embryos injected with 10 ng of a standard control MO. Embryos right themselves and swiftly swam a short distance from the point of contact. 10.1523/JNEUROSCI.3881-16.2017.video.1

Movie 2. Abnormal touch response in Stage 28 *Xenopus tropicalis* embryos. Real-time video of the abnormal touch response in Stage 28 embryos injected with a total of 10 ng *n1-src* AMO a+d. Nonresponding phenotypes remain horizontal and moved slowly from the point of contact with uncoordinated twitching movements. 10.1523/JNEUROSCI.3881-16.2017.video.2

Movie 3. Normal touch response in Stage 41 *Xenopus tropicalis* embryos. Real-time video showing the normal touch response of Stage 41 embryos injected with 10 ng of a standard control MO. Embryos right themselves and swiftly swam a short distance from the point of contact. 10.1523/JNEUROSCI.3881-16.2017.video.3

Movie 4. Abnormal touch response in Stage 41 *Xenopus tropicalis* embryos. Real-time video of the abnormal touch response in Stage 41 embryos injected with a total of 10 ng *n1-src* AMO a+d. Nonresponding phenotypes remain horizontal and moved slowly from the point of contact with uncoordinated twitching movements. 10.1523/JNEUROSCI.3881-16.2017.video.4

### *n1-src* knockdown disrupts primary neurogenesis

To ascertain which neurons in the touch reflex are affected by *n1-src* knockdown, we next investigated the early development of the primary nervous system. During primary neurogenesis, the motor neurons, interneurons, and Rohon-Beard sensory neurons differentiate to form medial, intermediate, and lateral columns respectively on either side of the neural plate midline. These columns of differentiating neurons are separated by nondifferentiating, proliferative progenitors and can be identified by expression of the neuronal-specific *tubb2b* gene (Chitnis et al., 1995). Figure 4*F* shows differentiating primary neurons visualized by *in situ* hybridization for *tubb2b* mRNA. Unilateral injection of AMO a+d resulted in a statistically significant reduction (χ^2^ = 44.922, df = 1, *p* = 2.05 × 10^−11^) of *tubb2b* expression in all three columns relative to the uninjected contralateral side (50 of 55; 90.9% abnormal) compared with embryos unilaterally injected with a control MO (8 of 39; 20.5% abnormal). Thus, we concluded that rather than regulating the development of specific subsets of neurons, *n1-src* is required for neurogenesis of all *tubb2b*-positive neurons in *Xenopus* primary nervous system development.

## Discussion

### The activity of amphibian and mammalian n1-src is conserved

The alternative splicing of neuronal src isoforms alters the ligand-binding specificity of the C-Src SH3 domain and the catalytic activity of its kinase domain (Brugge et al., 1985; Keenan et al., 2015). These differences are believed to underpin the reported differential activity of neuronal Srcs. The position of the N1-specific insertion into the SH3 domain of C-Src is conserved between amphibians and amniotes, and we investigated whether the differential biological activity of n1-src isoforms has been conserved in amphibians. We find that, unlike C-Src, both *Xenopus* n1-src and mammalian N1-Src, despite different SH3 inserts (5 vs 6 aa in amphibians and mammals respectively), are able to induce neurite-like processes when transfected into COS-7 cells. In keeping with this, it has been shown previously that N1-Src overexpression in *Xenopus* A6 epithelial cells induced neurite-like processes, in contrast to the rounded phenotype of C-Src-transfected cells (Worley et al., 1997).

### n1-src expression correlates with primary neurogenesis

A previous study indicated that the expression of the *n1-src* isoforms of the tetraploid amphibian *Xenopus laevis* are initiated by midneurula Stage 15 (Collett and Steele, 1992). We find that in the diploid amphibian *Xenopus tropicalis*, there is low-level maternal *n1-src* expression from the start of development, and, in contrast to the previous study, activation of zygotic *n1-src* expression is initiated as early as middle to late gastrula stages, and by early neurula stages expression is restricted to the open neural plate.

The period from late gastrula to early neurula is a key phase in the development of the primary nervous system, a simple functional nervous system, characteristic of anamniotic aquatic vertebrates, including fish and amphibians (Hartenstein, 1989). Differentiation of primary neurons enables the early development of motility, and helps embryos avoid predation in an aquatic environment. Primary neurons begin to differentiate at open neural plate stages in *Xenopus* embryos and primary neurogenesis continues through neurula and early tailbud stages (Schlosser et al., 2002). In keeping with our findings that *n1-src* expression is initiated during the gastrula stage, a subsequent study by Collett and Steele (1992) showed that *n1-src* expression is rapidly activated in competent gastrula dorsal ectoderm by endogenous neural-inducing signals and by the neural-inducing activity of the phorbol ester TPA in the absence of protein synthesis.

### Abnormal neural development in n1-src knockdown embryos

We present the first analysis of the consequences of blocking the splicing events required for the expression of *n1-src* during vertebrate development. An advantage of our approach is that the morpholinos ablated *n1-src* expression while *c-src* expression was unaffected. N1-src knockdown caused striking behavioral, morphological, and neuronal phenotypes in the *Xenopus* embryo. At the larval stage, we found that AMO-injected embryos exhibited a severely abnormal locomotor response to touch stimuli, with many observed to twitch or spasm following the touch. The *Xenopus* touch reflex and subsequent swimming circuits involve the coordination of sensory neurons, interneurons, and motor neurons (Roberts et al., 2010). In Stage 14 embryos, and in keeping with the widespread expression of *n1-src* in the neural plate, we found that the columns of differentiating *tubb2a*-positive neurons that subsequently form the touch and swimming circuits are reduced or absent. Due to the labile nature of the morpholinos, we predict that n1-src expression will slowly return, leading to a delay and perturbation in primary neurogenesis that generates the aberrant circuits observed in the larval embryo. The reduced touch response could also result from a defect in myelination, a process that begins at approximately Stage 42 (Yoshida, 1997). Furthermore, oligodendrocytes arise from the same precursors as motor neurons (Park et al., 2002). However, our observation that both Stage 28 and Stage 41 embryos exhibit the same defects rules out myelination as the sole cause of the phenotype.

Morphologically, n1-src-knockdown embryos exhibited a loss of retinal pigmentation and a kinked tail. The optic stalk, retina, and retinal pigmented epithelium develop from an outpocketing of the diencephalon (Fuhrmann et al., 2014). Therefore, the loss of eye pigmentation in n1-src-knockdown embryos may indicate a common role for n1-src in regulating the differentiation of cells derived from the neuroepithelium. The morphogenesis of the vertebrate main body axis involves coordinated cell movements in the axial mesoderm and the neuroepithelium (Nikolopoulou et al., 2017). Posterior axial defects have been observed in embryos in which normal convergent extension within the neuroepithelium has been inhibited through interference with components of the planar cell polarity signaling pathway (Goto and Keller, 2002). We speculate that the kinking of the posterior axis observed in n1-src-knockdown embryos arises through deregulation of the process of convergent extension, which drives elongation and narrowing of the neuroepithelium and reflects abnormal signaling within the neuroepithelium in the absence of n1-src activity.

At present we are unable to say with any confidence where n1-src functions in the pathway leading to neuronal differentiation. However, *n1-src's* general neural expression is similar to that of a group of neural stabilization genes, including members of the Sox, Zic, and Iroquois families (for review, see Moody and Je, 2002). These code for transcription factors and, as is the case with *n1-src*, several of these genes are expressed in response to neural induction. Neural stabilization genes have multiple overlapping functions, providing a link between the signals that induce the neural plate and the hierarchy of proneural and neurogenic genes required for neuronal specification and differentiation. Thus, early expressed neural stabilization genes have roles in regulating the competence of ectodermal cells to respond to neural-inducing signals, and later expressed ones regulate the progression from neuronal progenitor to differentiated neuron (Moody and Je, 2002). Current evidence is suggestive of a role for n1-src in the process of neural stabilization. However, future studies will be required to investigate the regulatory interactions between the n1-src kinase and known components of the vertebrate neurogenic pathway.

### N1-Src function and neurogenesis in higher vertebrates

Our data show that following primary neurogenesis, *n1-src* expression falls, but is again elevated during a second phase of neurogenesis in late larval stages. A primary nervous system is absent in amniotes, and it is the later phase of secondary neurogenesis in the closed neural tube that is more akin to the neurogenesis of amniotes, including mice and humans (Wullimann et al., 2005). A connection between neuronal differentiation and N1-Src function in amniotes is supported by an analysis of N1-Src (also termed pp60^+^) activity in the developing mouse brain, which showed a rapid increase in N1-Src activity at embryonic day (E) 12 and which peaks at E18, when increasing numbers of neuroblasts are exiting the cell cycle and differentiating (Wiestler and Walter, 1988). Furthermore, cultured neurons of the rat striatum contain little detectable N1-Src activity. However, neuronal differentiation induced by serum starvation leads to a sevenfold increase in N1-Src activity relative to C-Src (Cartwright et al., 1987). Similarly, embryonic carcinoma cells treated with retinoic acid to induce neuronal differentiation express increased levels of N1-Src (Lynch et al., 1986), and there is an increase in both N1-Src and N2-Src expression during differentiation of the neuroblastoma cell line LAN-5 (Matsunaga et al., 1993).

We present evidence for an early role for n1-src in neural development regulating the transition from neural progenitors to differentiated neurons. However, there is also evidence that N1-Src has roles regulating the cellular architecture and morphogenesis of neurons. Transgenic mice expressing N1-Src in Purkinje neurons of the cerebellum display defects in migration and dendrite morphology, which might be linked to defects in microtubule structure (Kotani et al., 2007). Conversely, in *Xenopus laevis*, overexpression of mammalian N1-Src in the optic tectum enhanced axonogenesis of retinal progenitors. Thus, n1-src is likely to have multiple roles in neural development regulating neuronal specification and morphogenesis.
